# Primary palliative care team perspectives on coordinating and managing people with advanced cancer in the community: a qualitative study

**DOI:** 10.1186/s12875-018-0861-z

**Published:** 2018-11-20

**Authors:** Julia Hackett, Lucy Ziegler, Mary Godfrey, Robbie Foy, Michael I. Bennett

**Affiliations:** 1 Martin House Research Centre, Social Policy Research Unit, University of York, York, YO10 5DD UK; 2 Academic Unit of Palliative Care, Leeds Institute of Health Sciences, University of Leeds, Level 10 Worsley Building, Clarendon Way, Leeds, LS2 9NL UK; 3 Academic Unit of Elderly Care and Rehabilitation, Bradford Institute for Health Research, Temple Bank House, Bradford Royal Infirmary, Duckworth Lane, Bradford, BD9 6RJ UK; 4 Academic Unit of Primary Care, Leeds Institute of Health Sciences, University of Leeds, Level 10 Worsley Building, Clarendon Way, Leeds, LS2 9NL UK

**Keywords:** Primary care, Palliative care, Gold standards framework, Qualitative research, Patient care

## Abstract

**Background:**

Primary health care teams are key to the delivery of care for patients with advanced cancer during the last year of life. The Gold Standards Framework is proposed as a mechanism for coordinating and guiding identification, assessment, and support. There are still considerable variations in practice despite its introduction. The aim of this qualitative study is to improve understanding of variations in practice through exploring the perspectives and experiences of members of primary health care teams involved in the care of patients with advanced cancer.

**Methods:**

Qualitative, semi-structured interviews**,** focus groups, and non-participatory observations involving 67 members of primary health care teams providing palliative care. Data were analysed using a grounded theory approach.

**Results:**

We identified distinct differences in the drivers and barriers of community advanced cancer care coordination, which relate to identification and management, and access to effective pain management, and go some way to understanding variations in practice. These include proactive identification processes, time and resource pressures, unclear roles and responsibilities, poor multidisciplinary working, and inflexible models for referral and prescribing. These provide valuable insight into how professionals work together and independently within an infrastructure that can both support and hinder the provision of effective community palliative care.

**Conclusions:**

Whilst the GSF is a guide for good practice, alone it is not a mechanism for change. Rather it provides a framework for describing quality of practice that was already occurring. Consequently, there will continue to be variations in practice.

**Electronic supplementary material:**

The online version of this article (10.1186/s12875-018-0861-z) contains supplementary material, which is available to authorized users.

## Background

Good provision of palliative care is a continuing clinical priority worldwide. People with advanced cancer are living longer and illness trajectories are changing [1, 2]. Prevalence of symptoms, such as complex pain, are likely to increase [3], requiring more input and support from a range of health professionals over longer periods of time. Receiving care at home is of great importance for most patients [4]. In the UK, primary health care teams (PHCTs) are multidisciplinary teams which are intended to provide the majority of palliative care to community based patients in the last year of life (see Table 1 for how the organisation of care across the PHCT is configured). High quality community-based palliative care requires good multidisciplinary teamwork [5–7] and close working relationships [8].

**Table 1:** The organisation of care across the PHCT

PHCT member	Role with patients with palliative care needs	Timing and type of involvement	How is involvement initiated	Method of involvement
GP	Provide general palliative care Assess patients’ needs Prescribe and manage medications Identify patients approaching end-of-life Care planning and anticipatory prescribing Manage and coordinate end-of-life care	Prior to diagnosis Continuous however during period where patient is receiving treatment may be intermittent until later stages	Patient presents to GP Referral from oncology	Appointments in surgery Home visits Occasional phone calls to patient and family
District nurse	Provide general palliative care alongside GP, i.e.: management, coordination, and orchestration of services to enable good home care for dying patients Physical nursing needs, i.e.: wound management, continence care, catheter care, medication and syringe drivers	Last few weeks/days of life Often receive a referral soon after diagnosis of advanced cancer so will have initial meeting and then intermittent contact until later stages Continuous involvement in last few weeks/days of life	Referral from GP, oncologist, community matron, joint care manager, clinical nurse specialist	Always home visits Sometimes phone calls to patient and family
Clinical nurse specialist	Provide specialist psychological and physical symptom management that	Can be from diagnosis of advanced cancer Intermittent	Referral from GP, district nurse, oncologist Complex needs that cannot be managed by the GP and district nurse	Always home visits Often phone calls to patient and family
Community matron	Provide care and support to people with long-term chronic conditions to keep patients as healthy as possible and living independently Only involved if patient has a long-term chronic condition and cancer	From diagnosis of chronic condition Continuous	Referral from GP, district nurse, hospital team	Always home visits Sometimes phone calls to patient and family
Joint care manager	Provide a service to adults aged 65 years and over with complex health and social care needs and adults of all ages who have been identified as eligible for NHS Continuing Healthcare funding Assess health and social care needs; plan, coordinate, and review services required	Discharge from hospital At home but at risk of being admitted to hospital/care home when don’t need to be In a Community Intermediate Care bed or at home with services to help you with personal care from Leeds Community Healthcare NHS Trust and need ongoing care Continuous	Referral from any health or social care professional	Home visits
Complex and palliative continuing care service	Provide bespoke packages of care to fast-track patients with highly complex continuing care needs	Last few days of life Continuous	Referral from district nurse	Home visits

The Gold Standards Framework (GSF) describes and brings together a number of evidence-based principles of practice as a guide for the care of palliative patients and their families [9]. It was intended to help PHCTs identify, assess, and support patients with palliative care needs. It can be incorporated into general practice infrastructures at various levels (Table 2). Practices which achieve level 1, the maintenance of a register and regular palliative care meetings, are currently financially rewarded through the Quality and Outcomes Framework (QOF) [10]. Whilst the GSF is thought to have contributed to better-quality care, for example: in improving early identification of patients, there is still much room for improvement [11, 12].

**Table 2:** The four levels of adoption of the GSF

Level 1	*Communication*	Compiling and maintaining a supportive care register to record, plan, and monitor patient care
	*Coordination*	Having a nominated coordinator to oversee implementation and maintenance of the framework
Level 2	*Control of symptoms*	Patients’ symptoms, problems, concerns are assessed, recorded, discussed and acted upon to an agreed process. Advanced care planning tools are recommended
	*Continuity*	Systems to ensure continuity of care delivered by inter-professional teams and out-of-hours providers are used. Anticipatory care in place to reduce crises and inappropriate admissions
	*Continued learning*	Commitment to learning about end-of-life care and developing action plans to meet identified learning needs. Reflection on past events, what went well and why, and what did not go well and why
Level 3	*Carer support*	Work in partnership with carer and assess and support their needs for emotional, practical, and bereavement support
	*Care of the dying*	Appropriate care provided in the last days of life
Level 4	*Sustain, embed and extend improvements in end-of-life care*	Sustain and build on all developments as standard practice. Develop a practice protocol and extend to other settings, e.g.: care homes, non-cancer, ACP

Although the majority of practices in England have adopted the GSF in some form, to at least level 1, there are well-recognised variations in practice [6, 13] associated with shortcomings in the processes for coordinating, managing, and providing effective care [14, 15]. Whilst, there has been evaluation and measurement of the effects of using GSF at every stage, it is hard to describe exactly what the benefits have been for those who have used it and whether it has facilitated good multidisciplinary working. Previous research has shown that there is considerable variation between practices in the effectiveness of interprofessional communication [10]. Practices categorised as ‘high performing practices’ are reported to display a clear shared purpose among staff for palliative care, whereas those categorised as ‘minimal performing practices’ demonstrate little utilisation of basic processes recommended in the framework and deficiencies in interprofessional communication. Effective primary palliative care requires good team relationships and communication [6]. We therefore explored the perspectives and experiences of PHCT members who are involved in the multidisciplinary care of patients with cancer to see whether these can improve understanding of variations in practice.

## Method

### Design and participants

We conducted semi-structured interviews and focus groups with purposively selected PHCT members and non-participatory observations of multidisciplinary GSF meetings. We explored the perspectives and experiences of PHCT members who provide care to people with advanced cancer in the community to see whether these can improve understanding of variations in practice. For the purpose of this study, care provided in the community relates only to people with palliative care needs being cared for in the home. Advanced cancer is defined as active and non-curative. The research was conducted in Leeds, UK and was part of a research programme on the management of advanced cancer pain.

### Data collection

First, L.Z. (an experienced psycho-oncology researcher) conducted five single-professional focus groups with key community professionals who covered the whole city: clinical nurse specialists (CNSs), community matrons, joint care managers, members of the complex and palliative continuing care service (CAPCCS), and GPs. These explored experiences of coordinating and managing people with advanced cancer in the community using a topic guide, which was based upon a review of the current literature at that time (Additional file 1). This provided a comprehensive picture of practice across the city and suggested that effective cancer pain management extended beyond individual professional practice, skills, or experience to broader barriers related to the organisations and systems within which professionals work.

Subsequently, as a sampling strategy, we asked practice managers and GP leads across all general practices in Leeds a series of questions based on the levels of the GSF to determine practice approaches to coordination and management. This allowed us to identify different models of advanced cancer care coordination. We purposively selected six of these practices, representing two different approaches to care, which we categorised as high and minimal performers according to their responses, and contacted them by letter/follow up telephone call. Three responded and gave written consent to allow non-participant observations of multidisciplinary GSF meetings (one at each practice). The aim of these observations was to build up a picture of how care of patients with advanced cancer is routinely organised, how decisions are made about assessment of pain, how this is communicated between professionals, how strategies for management are devised and which professionals are seen as key in delivering support and care. Notes on these aspects and non-verbal communication were taken by the observer (J.H., an experienced health sciences researcher) during these meetings and subsequently expanded. Interviews were conducted by J.H. with individuals identified during GSF meetings as being most involved in coordinating care. Interviews explored experiences of managing people with advanced cancer in the community using a topic guide, which was based upon a second review of the literature and findings from the initial focus groups (Additional file 2). Informed written consent was obtained from all individual participants included in the study. All focus groups and interviews took place in participants’ place of work, during working hours, no one else was present. Participants were not known to the researchers prior to the study.

### Data analysis

Interviews and focus groups were audio-recorded and transcribed verbatim. Data were collected over two periods, from June to August 2010 and April 2013 to September 2014. Each participant was given a unique identifier to maintain confidentiality.

We adopted a grounded theory analytic approach [16, 17] as it provides an approach to construct theory driven understanding of health professionals behaviour and the factors that influence it. This combined concurrent data collection and analysis with modification to the topic guide to pursue emerging lines of enquiry. Debrief meetings for researchers took place after each interview providing space to reflect on the interview process and explore initial ideas. Further steps carried out by authors, separately and together, were familiarisation of the whole data set through multiple readings of transcripts, open and focused coding, memo writing and engagement with the literature, to facilitate the development of categories and concepts. Constant comparison and searching for negative cases were used throughout, whereby data segments and the developing codes and categories were compared both within cases to identify the sequencing of events, and how these were understood and acted upon, and between cases to examine variations between participants. Throughout data collection and analysis, data, codes and concepts were discussed within the research team (of varied disciplinary backgrounds: psychology, health sciences, sociology, and academic palliative care medicine) and with the wider steering group. The latter included patient representatives, clinicians and academics.

## Results

In total, 67 health professionals took part. Five single professional focus groups comprised 27 health professionals: six GPs, eight CNSs, five joint care managers, four members of the Complex and palliative continuing care service, and four community matrons. Twenty-four general practice managers and/or GP leads responded to a series of questions about practice approaches to coordination and management of people with advanced cancer. Three practices were then selected (Tables 3 and 4) for non-participatory observation, involving a total of 32 health professionals. Eight interviews were then conducted with professionals: three GPs, three CNSs, and two district nurses. Mean focus group length was 31mins (range: 27 to 36mins) and mean interview length was 52mins (range: 27 to 64mins). Total observation time was 145 mins.

**Table 3:** Practice characteristics for practices participating in observations and related health professional interviews

	Practice		
	A	B	C
List size range	5000–10,000	20,000–25,000	10,000–15,000
Deprivation decile	6	8	9
Full time GP partners	4	6	4
Part-time GP partners	1	4	4
Full time GP salaried	0	2	0
Part-time GP salaried	0	5	1
QOF achievement palliative care (%)	100	100	100
Number of staff at GSF meeting	10	13	9
Number of staff interviewed	3	2	3

Note: deprivation decile scores, 1 most deprived to 10 least deprived

**Table 4:** Levels of GSF adoption for practices participating in observations and related health professional interviews

Key tasks	Practice A	Practice B	Practice C
*Communication*	Set up register Regular GSF meetings	Set up register Less regular GSF meetings	Set up register Regular GSF meetings
*Co-ordination*	Lead GP has special interest and is responsible for coordinating meeting and register DN input from DN team, no specific lead DN	Lead GP has special interest and is responsible for coordinating meeting and registerLead DN for practice	GP lead has no ownership, CNS is responsible for coordinating meeting and highlighting patients for register Lead DN for practice
*Control of symptoms*	Confident in symptom control and pool knowledge with other services Do not routinely use assessment tools	Lack of confidence in symptom control, but shared care with/supported by CNS and DN services Use assessment tools	Lack of confidence in symptom control and leave care to other services Lack of use of assessment tools
*Continuity of care*	Shared care with secondary care	Shared care with secondary care	Lack of continuity of care with secondary care, will not take responsibility of care or participate in shared care
*Continued learning*	Use of significant/after death analysis Identify and address knowledge gaps	Use of significant/after death analysis but infrequency of meetings impinges on this	Do not carry out continued learning unless instigated and led by CNS
*Carer support*	Carer support Extend care into bereavement phase	Carer support evident but infrequency of meetings impinges on this Extend care into bereavement phase	Carer and bereavement support left to CNS and not discussed within practice
*Care in the dying phase*	Involved in dying phase	Involved in dying phase	Reluctance to engage in dying phase

There were distinct differences in the drivers and barriers within the two models of community advanced cancer coordination. Each practice provided the system within which examples of multidisciplinary working, implementation of policy, and professional behaviours and attitudes could be mapped and subsequently compared. Drivers and barriers for these distinct models of operation include proactive identification processes, time and resource pressures, unclear roles and responsibilities, poor multidisciplinary working, and inflexible models for referral and prescribing. These are now presented within two key themes: identification and management, and access to effective pain management, with three subthemes: prescribing restrictions, home visits, and referrals.

### Identification and management

A proactive approach, with early identification of and care planning for patients, was key to coordination and management. Proactivity demonstrated whether practices viewed people with advanced cancer as different, or not, from the general practice population and therefore requiring specific, flexible input. High performing practices, such as A and B, adopted formal proactive processes to ensure identification and monitoring of patients who were not previously known to GPs. They would initiate contact and, in doing so, commence assessment, monitoring and management of pain.

If I receive a letter from the hospital that tells me somebody’s got a new diagnosis, I make contact. Then the obvious triggers like multiple hospital admissions, we look at that, and because of the evidence you’ll identify your 1% that way so if somebody’s bouncing in and out of hospital… Over time we’ve found that the register’s grown quite significantly, so we try to just discuss patients who are at the amber to red end of the traffic light system. (GP 8, Practice B)

These practices were engaged with and had ownership of the GSF, demonstrated through their management of the palliative care register and GSF meetings, and had clear roles and responsibilities within the primary palliative care team. For example: in practices A and B, the palliative care lead GP, both of whom had a special interest in palliative care, was the nominated coordinator of the all work relating to the GSF, they led the meeting, and GPs generated and maintained the register personally, ensuring that all eligible patients were recorded. Patients mentioned at the meeting by the CNS or district nurse were already known to GPs and new referrals were noted down and recorded. Through this system they not only had ownership of the GSF, but achieved level 1. They also adopted flexible working practices, for example: a buddy system was used, whereby patients had a second named GP to account for periods of annual leave or part-time working. Meetings consisted of discussions on patients’ status, managing needs, bereavement support, and significant event or after death review to re-appraise appropriateness of response. Therefore achieving levels 2 and 3 of the GSF.

Practice C did not differentiate between people with advanced cancer and other patients, did not regard them as requiring any special input, and therefore failed to adopt any flexible working practices. Observations highlighted a lack of ownership over the GSF and unclear roles and responsibilities within the primary palliative care team, for example: although they had a nominated coordinator to oversee all work relating to the GSF, this was the CNS, and not the palliative care lead GP, as a result the CNS led the meeting and identified patients for the register. Patients identified by the CNS and DN were not already known to the practice. There were no formal, proactive processes for identifying patients embedded as standard practice.

I try and actively encourage them to look at the palliative care register in terms of getting additional people on. They really don’t engage with that. I’ve asked several times “do you know if we’ve any other patients to discuss?” and they don’t have a system. They do have a list, I’m just not sure how up to date that is and they’ve said they don’t want a long list, there’d be loads of patients. And we’re not really meant to lead the meeting, it should be the GP. I’m meant to be there as an additional person to contribute, and they just listen to me and chip in, it’s meant to be the other way round. (CNS 9, Practice C)

Only patients already registered with the palliative care service were on their register. Although discussions about updates on patients’ status and how to manage needs occurred, these were instigated by the CNS and district nurse, who felt these discussions were time limited and not seen as a priority. Consequently, although aspects of levels 2 and 3 of the GSF were covered, the impetus for them was led by specialist practitioners, demonstrating a lack of ownership from the practice.

### Access to effective pain management

Patient access to effective cancer pain management was highly variable and influenced by professional priorities and complex practice level policies. The levels to which the GSF was adopted led to variations in effective pain management activities, in particular: prescribing restrictions, home visits, and referrals.

#### Prescribing restrictions

Many general practices operate a practice level policy whereby non-cancer patients are required to wait 48 h before prescriptions can be collected, however such policies may be flexible based on patient need. Practice C was relatively inflexible in its prescribing policy for people with advanced cancer. This limited the potential for a timely response to changing pain needs. The importance of this barrier is not only related to the need to offer fast, accessible services to cancer patients; but to the fact that some cancer patients only visit their practices to obtain repeat prescriptions and this represents their only encounter with primary care. This can influence their confidence in, and opinion of, their GP when care is transferred at a later stage of their illness.

I had a very elderly lady on absolutely huge doses…but she still needed quite a lot of breakthrough analgesia, I went to see her and I rang the GP. “This is what I need, I need it today…”, “Yes, it will be ready”. When her husband went to pick it up, it wasn’t ready and they said to him, “No, you will have to wait ‘til Monday now”. And then over that weekend they tried to struggle through with the OxyNorm and he had to get the emergency doctor out and his family had to drive round looking for a chemist that was open for these drugs. (CNS 8, focus group)

They also maintained a tight rein on prescribing budgets and minimised costs wherever possible. Medications prescribed on discharge from hospital were changed by GPs to explore whether a cheaper alternative might be effective. In an attempt to minimise potential wastage, patients were given limited supplies of anticipatory drugs. These universal cost saving prescribing practices were described in the context of patients with advanced cancer and demonstrated that despite such a diagnosis, the policy was not modified. Advanced cancer patients were treated in the same way as other patients in judgements made about prescribing.

I reckon if someone isn’t long for this world I would give them two vials of something to get them through the weekend and I’ll see if they’re still here on Monday. And then I’m more than happy to give them more. Otherwise I think there’s too much wastage in the NHS. (GP 12, Practice C)

In contrast, others believed that there was no justification for trying to limit costs, particularly given the limited length of time the patient would require the drug and that the greatest costs to the NHS would be having to fast track a patient or an unplanned hospital admission. The ethos underpinning policy and practice here was that palliative care requires effective management of symptoms. In the multidisciplinary focus groups and interviews with CNSs, they agreed that patients with advanced cancer should be prioritised in terms of access to the most effective and tolerable pain control irrespective of cost. This suggests that minimising the cost of analgesia in advanced cancer patients is not a high priority for all and in this instance, the policy appeared to be generated and maintained at a practice level.

I think cost does have a bearing, whereas I probably don’t give a great deal of consideration to costing. I know that some GPs do, some of the drugs they’re maybe not too keen to try and some will even suggest that they would prefer to go for something else as an alternative and they will mention costing. (CNS 7, focus group)

In instances where GPs were known to be difficult to work with, poor multidisciplinary working was evident as CNS’s recounted times when they went around them instead of negotiating with them.

There are always some GPs that you feel might be less amenable to prescribing for various reasons, if you felt that the GP wasn’t willing to do what you thought was appropriate and couldn’t give you a valid reason in your mind as to why, then in essence we would bypass the GP if we felt we couldn’t negotiate with them. (CNS 8, focus group)

However, this then has repercussions for overall coordination and management, particularly monitoring of symptoms and care planning.

#### Home visits

Patient access to effective cancer pain management was highly variable and influenced by professional priorities and complex practice and service level policies and pressures. One such practice-level policy relates to GPs undertaking home visits, especially within the context of perceived increasing pressures and demands on time. Within practices A and B, GPs initiated and negotiated their involvement and viewed themselves as being involved in patient care from diagnosis to death.

I think most people who are having palliative care at home will see their GP as the coordinator of that care. A GP is key to these patients, I do think we’re very important. We ask them what they want, I don’t force the issue, you know you can say to me if you want me to, “I would happily see you on a monthly basis” or whatever. In their last weeks to months of life when you have known them very well, I think you are a befriender as much as a clinician. (GP 8, Practice B)

In contrast, practice C were reactive in their approach to care, evidenced by their lack of initiating or actively maintaining involvement with patients. Home visits were only undertaken in exceptional circumstances and were identified as a nurse’s responsibility rather than the GP’s.

We run more of a demand led system so it’s up to the patient to ask, to make appointments, we don’t have capacity really, if patients can come to the surgery we encourage it…Once they get more poorly, I think the district nurses and the Macmillan team take over more, we have more to do with people when they’re able to come to see us. (GP 7, Practice C)

Others highlighted home visits as a high priority, with GPs and nurses proactively advocating for joint visits as a key component of effective pain assessment and management. Sometimes it was necessary to spend time with patients and their relatives, perhaps over consecutive days, to fully understand the nature of their symptoms. This also contributed to building a relationship, facilitating disclosure of the reality of patients’ pain, and providing psychological support.

I think personally it’s nice to see them face to face. It’s a lot nicer to see them in their own home, you probably feel like you’ve got more time, they’re feeling more comfortable and a bit more secure to talk about things that they’re not happy to talk about, it’s more on their terms then. (GP 8, Practice B)

#### Referrals

GPs were highlighted by CNS’s as the main referrer to their service. However, due to a lack of proactive identification processes, instances were recounted where patients had been missed and therefore not referred.

Quite often they’ll say on the letters ‘may be worth involving palliative care’ but it still relies on the GP seeing and deciding to do that. I’m thinking of that patient we saw at home, when you got the information from the oncologist the last two or three letters to the GP had suggested that there should be a referral to palliative care and the GP just hadn’t done it. (CNS 4, focus group)

Poor multidisciplinary working was also evidenced by CNS’s describing cases where patients were referred to their service without their GP knowing.

By far the majority of the referrals come from GPs to the CNSs. There's occasional one that a consultant will refer direct to me from an outpatients and I will look at this and think why have you done that? (CNS 1, focus group)

This had consequences for patient access to other services, particularly district nursing, who are supposed to provide general palliative care alongside the GP. These services were then at a disadvantage as they have been brought in late to patient care.

It just doesn’t happen, we get the referral and then we then refer to the DNs. They’re a really vital role of that consistent support monitoring and holistic care for them and we’re coming in focussing on their specialist needs, not their general. If they don’t have that district nurse, they often end up in crisis and then just come straight to us. Whereas, any district nurses then are deskilled in the palliative care. (CNS 9, Practice C)

Consequently, this lack of coordination and poor multidisciplinary working resulted in instances where there was no coherent pathway for patients to navigate through the services.

## Discussion

We explored the perspectives and experiences of PCHT members who are involved in the multidisciplinary care of people with advanced cancer, to see whether these improved understanding of variations in practice. Within our research, Practices A and B were identified as high performing sites, whilst Practice C was identified as a minimal performer. Where this paper adds knowledge is that there were distinct differences in the drivers and barriers within these models of community advanced cancer coordination which help explain variations in practice. Practices A and B adopted formal proactive processes for identifying advanced cancer patients. They had clear roles and responsibilities with their primary palliative care teams which enabled good multidisciplinary working; and adopted flexible approaches to care, in particular evidenced by their attitudes to referrals, home visits, and prescribing. Within these practices, we identified that if professionals adopted a flexible approach to care then the scope to deliver effective individualised pain management was enhanced. Practice C however, were reactive in their approach to coordinating and managing care for these patients. There were no formal mechanisms in place for identifying patients; unclear roles and responsibilities within the team, which impacted upon multidisciplinary working; evidence of time and resource pressures at a practice level; and inflexible models for referrals and prescribing. This reactive, inflexible, and untimely approach to care meant that individualised care was difficult to achieve.

The extent to which a practice subscribes to a palliative care philosophy appeared to be fundamental to the provision of effective advanced cancer care coordination and management. We demonstrate how professionals within a multidisciplinary team work within an infrastructure that can both support and hinder the provision of effective community palliative care. Practice C operated within an ethos that did not differentiate the specific needs of people with advanced cancer and this inhibited a flexible approach to pain management. Adopting a universalist approach with people with advanced cancer had unintended consequences for coordination and management. The policies and procedures which they adhered to appeared to provide a structured mechanism for decision making in relation to pain management and diverted practice away from the subjective and interactive processes related to pain management evident in Practices A and B. However, when such a policy is formalised within a practice setting, the scope for individual professional perspectives and subsequent variation in practice becomes limited, even when the professional was not part of the devising body. In addition, the operation of inflexible prescribing restrictions, is a policy that could result in unintended consequences that are potentially more costly to the NHS, such as unplanned hospital admissions. Practice C illustrates how a not uncommon set of external and internal constraints [18] can have unintended consequences for pain management. Any strategy to support practices in improving pain management must be informed by an understanding of such constraints.

Challenges to effective multidisciplinary team working are evidenced by nurses’ accounts of the difficulties in working with GPs, reporting power relationships, and implicit and explicit rules governing the process of inter-professional work [6, 18]. These dynamics sit at the core of providing effective primary palliative care. Professional identities and organisational structures affect coordination and management because these are key aspects of effective teamwork. The GSF may have been intended to provide a framework to guide care and provide a toolkit for the coordination and management of advanced cancer care, however our data illustrates that, despite the financial incentives associated with it, it is inadequate in recognising the complexity of practice and implementation of change. Instead of providing a mechanism for change, we suggest that it provides a framework for describing quality of practice that was already occurring. It is a guide for good practice, but fails to describe an implementation approach, therefore cannot itself change practice.

In high performing practices, GPs were proactive in identifying and coordinating care in order to aim for continuity with patients [19, 20]. They were engaged with and took ownership of the GSF and had clear roles and responsibilities. Although engagement and continuity are key, the workload of primary care is growing [20, 21], with more GPs are working part-time, and out-of-hours care more frequently occurring with health professionals who are unfamiliar with the patient [22]. Our findings show how these developments can be overcome by providing proactive care and putting flexible systems in place to take account of these changes, for example: having a second named GP to cover periods of annual leave or part-time working. Future developments must recognise the changing landscape of primary care to enable adaptation.

Timely referrals were highlighted as enabling professionals to develop relationships with patients and their families earlier, enhancing the ability to deliver effective individualised patient care and enabling continuity [6]. The current focus of the GSF in the last year of life doesn’t take account of the shifting trajectory of advanced cancer, including the increasing need for input and support over longer periods of time [1]. We question how care should be initiated and coordinated when different members of the PHCT enter and exit patient care at different points, therefore have different levels of engagement, and view the meaning of palliative care from the perspective of their input. Standardised definitions of roles and responsibilities are needed [20, 23].

The way professionals, policies, and services within the UK primary care system interact is dynamic and complex, with many aspects of exactly how this occurs remaining unclear. This lack of clarity is likely to be due to the considerable variability in how the three components of the system interact, specifically the variability in the level of engagement between: generalists and specialists; professionals and patients; and professionals, policies, and service level initiatives. This means that although we are beginning to understand the component parts of this system, we do not fully understand the whole. For example, one of our study practices clearly recognised or perceived strong pressures to control costs and demand, and is unlikely to be atypical in doing so. This has implications for developing and targeting interventions. The recognition that advanced cancer coordination and management cancer-pain management in primary care occurs within a multidisciplinary team suggests that an intervention to improve this that was embedded at a professional or service level alone will struggle to be effective. Further measures to improve continuity and coordination need to be developed through close working with a range of practices, with varying abilities to respond to clinical policy frameworks.

### Strengths and limitations

Exploring the involvement of all members of the PHCT, allowed us to gather a wide range of views and subsequently focus on those most involved in care. A limitation is it took place in one UK city and we were unable to recruit more practices to take part in the observations and associated interviews, particularly from more deprived areas. Future research could explore these drivers and barriers within a larger number of practices, representing a wider range of deprivation. Whilst this could be a potential limitation of the analysis, the themes that emerged concerning the organisation of care resonate with those reported more widely [24, 25]. We identified two key contrasting approaches, although these may not be the only models of advanced cancer coordination and management, and illustrate and highlight drivers and barriers that can shape variation in practice. Secondly, our study spanned a change in the structure of primary care, when clinical commissioning groups replaced primary care trusts. Local priorities may have changed, however this was not evident within our findings. The significance of our study is that it provides insight into specific practice cultural and organisational factors that shape interpretation of policies and subsequent practice.

## Conclusion

We identified distinct differences in the drivers and barriers within these models of community advanced cancer care coordination. These provide valuable insight into how professionals work together and independently within an infrastructure that can both support and hinder the provision of effective community palliative care. Whilst the GSF is a guide for good practice, it fails to describe an implementation approach, therefore is not a mechanism for change. Consequently, there will continue to be variations in practice. If general practices remain purely reactive in their approach to care, then this will have unintended consequences for coordination and management. Overcoming these issues is key to ensuring the provision of effective community palliative care.

## Additional files

Additional file 1: Focus Group Topic Guide. Topic guide for focus groups. (DOCX 13 kb)

Additional file 2: Interview Topic Guide. Topic guide for interviews. (DOCX 13 kb)

## Abbreviations

CAPCCS: Complex and palliative continuing care service
CNS: Clinical nurse specialist
GP: General practitioner
GSF: Gold Standards Framework
PHCT: Primary health care teams
QOF: Quality and Outcomes Framework
