# Supplementary material for: Primary palliative care team perspectives on coordinating and managing people with advanced cancer in the community: a qualitative study
Source: BMC Fam Pract. 2018 Nov 20;19:177. doi: 10.1186/s12875-018-0861-z (PMC6247763; doi:10.1186/s12875-018-0861-z)
Supplement: Supplementary file 1 — Focus Group Topic Guide. Topic guide for focus groups. (DOCX 13 kb) [file 12875_2018_861_MOESM1_ESM.docx]

**Additional File 1**

Focus Group Topic Guide

| **Section** | **Types of questions/prompts** |
| --- | --- |
| Background | Yourself and your work   - Length of time in service - Types of patients you see - How often - Setting - Other teams |
| Evaluating pain | Symptoms, features, behaviours of pain  Discuss pain with patients   - When - How   Roles in pain assessment and management   - When - How - Screening tool   Collaborating with other teams/professionals in coordinating care and managing pain  Referral patterns and criteria  Problems in detecting and managing pain   - Challenges - Time constraints - Confidence |
