# Supplementary material for: Primary palliative care team perspectives on coordinating and managing people with advanced cancer in the community: a qualitative study
Source: BMC Fam Pract. 2018 Nov 20;19:177. doi: 10.1186/s12875-018-0861-z (PMC6247763; doi:10.1186/s12875-018-0861-z)
Supplement: Supplementary file 2 — Interview Topic Guide. Topic guide for interviews. (DOCX 13 kb) [file 12875_2018_861_MOESM2_ESM.docx]

**Additional File 2**

Interview Topic Guide

| **Section** | **Types of questions/prompts** |
| --- | --- |
| Background and coordination | Types of patients you see  Register management   - Who oversees it - How - When do patients go on the register - How are they subsequently managed - Traffic light system |
| GSF meetings | GSF meeting attendance, frequency   - Purpose - Usefulness - Action planning - Decisions - Impact on patient care |
| Communication with other health professionals | Within and between meeting communication   - Which professionals - What method - How does this compare with other practices |
| Roles and responsibilities | Own role and of other services   - Level, timing, and continuity of involvement   Interaction between services |
| Control of symptoms | Assessment, monitoring, and management  Medication management  Patient attitudes towards pain/pain relief |
| Communication with patients | Who do patients speak to about what/why/when/how? |
| Advanced care planning and carer support | Professional involvement  Anticipatory drugs  End of life wishes  Bereavement support |
| Continued learning | Significant event or after death review |
